# An injectable hydrogel for localized sustained release of artesunate in postoperative fibrosis prevention after glaucoma filtration surgery

**DOI:** 10.1080/10717544.2026.2686511

**Published:** 2026-08-03

**Authors:** Guangshuang Tan, Lusi Zhang, Tong Wu, Jingyuan Liu, Ying Wu, Manyi Xiao, Cong Wang, Zheng Pan, Shutong Wang, Chunrun Chen, Shanshan Zhu, Zewei Wang, Boyan Ma, Datong Xu, Liyang Shi, Bing Jiang

**Affiliations:** a Department of Ophthalmology, The Second Xiangya Hospital, Central South University, Changsha, China; b Hunan Clinical Research Center of Ophthalmic Disease, Changsha, China; c College of Basic Medicine, Beihua University, Jilin, China; d College of Biology, Hunan University, Changsha, China

**Keywords:** Sustained-release hydrogel, glaucoma filtration surgery, fibrosis, artesunate, glaucoma

## Abstract

Glaucoma filtration surgery (GFS) is the definitive treatment for reducing intraocular pressure in patients with refractory glaucoma. However, postoperative scarring of the filtration tract is a major cause of surgical failure. Although artesunate (ART) exhibits significant antifibrotic efficacy, its clinical application is severely limited by poor solubility and a short half-life. This study designed and developed a new injectable hydrogel cross-linked by collagen and four-armed polyethylene glycol *N*-hydroxysuccinimide encapsulating ART (C-P@ART) to overcome the limitations of ART in ocular antifibrotic therapy and extensively examined its underlying mechanisms. Stable three-dimensional network structure, excellent biocompatibility, and sustained drug release characteristics of C-P@ART were validated through FTIR, rheological analysis, endotoxin-testing, scanning electron microscope (SEM), and *in vitro* and *in vivo* release assays. In a TGF-β1-induced fibroblast fibrosis model, C-P@ART significantly inhibited *α*-SMA expression concentration-dependently and exerted antifibrotic effects through dual regulation of TGF-β1/SMAD and PI3K/Akt pathways. Subsequently, we evaluated its antifibrotic efficacy in the rabbit GFS model, where the C-P@ART treatment group exhibited a significant reduction in postoperative collagen deposition and *α*-SMA-positive expression compared to the control group. C-P@ART inhibited NF-κB pathway activation and reduced inflammatory cell infiltration. In conclusion, these findings demonstrated that C-P@ART synergistically mitigates fibrosis and inflammation through sustained ART release, providing an innovative therapeutic strategy to enhance GFS outcomes.

## Graphical abstract

**Figure:**
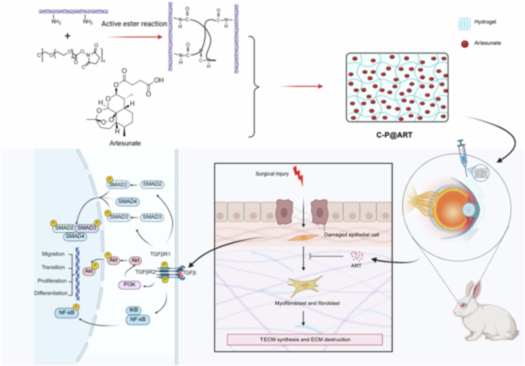

## 1. Introduction

Glaucoma is one of the leading causes of irreversible blindness globally (The 2021). It is estimated that the global population of patients with glaucoma will reach 112 million by 2040 (Li et al. 2016). Elevated intraocular pressure (IOP) is one of the significant risk factors for glaucoma (Casson 2022; Mathew and Barton 2011). However, its insidious onset results in many patients being diagnosed at advanced stages, where pharmacological or laser therapies exhibit limited efficacy. Therefore, surgical intervention is an essential therapeutic option for patients with poorly controlled IOP or advanced glaucoma (Zuo et al. 2018).

Glaucoma filtration surgery (GFS) aims to lower intraocular pressure by establishing an alternative drainage route. However, GFS has a high failure rate (15%–30%) due to excessive healing at the bleb and sclerostomy sites (Cabourne et al. 2015). Although intraoperative antimetabolite agents including mitomycin C and 5-fluorouracil effectively inhibit fibroblast proliferation, their nonselective cytotoxicity contributes to severe postoperative complications, including persistent hypotony, corneal endothelial toxicity, and filtering bleb leaks (Yamanaka et al. 2015). Accordingly, a new high-safety pharmaceutical capable of providing long-term antifibrotic effects is urgently needed to enhance GFS outcomes.

Our previous study has demonstrated that artesunate (ART) exhibits potent antifibrotic effects in GFS rabbits (Liu et al. 2023) with a higher level of biological safety than mitomycin when administered after surgery. The subconjunctival injection of ART is limited by its limited aqueous solubility (approximately 0.1 mg/mL in pure water, though increasing under weakly alkaline conditions) and its exceptionally short plasma half-life of only 2 to 10 min (Zhang et al. 2024a). Moreover, similar to antimetabolite agents, the transient exposure of ART fails to provide sustained inhibition of fibrotic cascades during the critical period of fibrosis (7–28 days postoperation) (Kim et al. 2015). A single administration of ART is inadequate to sustain its long-term antifibrotic efficacy; however, multiple dosing presents many clinical limitations, including patient compliance (Cate et al. 2012). Furthermore, the solubility of ART in conventional solvents poses a significant challenge for optimizing medication dosing to achieve efficacious antifibrotic outcomes. A new ART therapeutic strategy that minimizes systemic toxicity and achieves an extended duration of antifibrotic efficacy is required to address these limitations.

Injectable hydrogel systems have emerged as a transformative platform in biomedical applications because of their unique capacity for controlled drug release, targeted delivery, and excellent biocompatibility (Omidian and Chowdhury 2023). Extensive preclinical and clinical studies have demonstrated their therapeutic potential across various medical specialties, including orthopedics (Li et al. 2023), wound management (Wang et al. 2022a), pain control (Chen et al. 2022; Wu et al. 2025), and ophthalmology (Wang et al. 2023). These systems have been effectively utilized in ophthalmic applications, including soft contact lenses (Chau Thuy Nguyen et al. 2022), intraocular lenses (Cui et al. 2024), vitreous replacements (Su et al. 2011), and cell-based regenerative therapies (Li et al. 2022a). Compared to conventional therapies, temperature-sensitive and pH-sensitive injectable hydrogel systems are advantageous for GFS treatment. This approach aligns with the subconjunctival injection administration method in ophthalmology, which enhances the drug's solubility and stabilizes its half-life through sustained-release mechanisms, providing distinct pharmacokinetic advantages. Their compatibility with clinically approved pharmaceuticals facilitates rapid translation into clinical practice, potentially superseding conventional drug administration methods.

Postinjury fibrotic repair is a dynamic orchestration of overlapping inflammatory and fibrogenic phases (Lassance et al. 2018; Sampaio et al. 2021; Stepp and Menko 2021; Wilson 2022). In the GFS model, fibrosis occurs immediately after surgery and typically reaches its peak at day 7 (Wilson et al. 2018). Impaired extracellular basement membrane (EBM) regeneration triggers persistent transforming growth factor-β1 (TGF-β1) infiltration, which subsequently drives fibroblast-to-myofibroblast differentiation, excessive collagen deposition, and extracellular matrix (ECM) remodeling via both canonical SMAD2/3 (Caja et al. 2018; Inui et al. 2021; Lee and Massagué 2022; Zhang et al. 2020) and noncanonical PI3K/Akt signaling pathways (Zhang et al. 2023; 2024b). Notably, inflammation serves as the preliminary phase of the fibrotic process (Wen et al. 2022b). Activation of the NF-κB pathway triggers the transcription of proinflammatory cytokines, sustaining critical inflammatory-fibrotic crosstalk (Giridharan and Srinivasan 2018; Li et al. 2019; Li et al. 2022b). The resulting microenvironmental imbalance leads to the infiltration of inflammatory cells, which further secrete mediators that exacerbate the activation of pro-fibrotic signaling (Lafuse et al. 2020; Zhang et al. 2010).

Herein, we designed and fabricated a temperature- and pH-sensitive composite injectable hydrogel-based sustained-release drug delivery system for ART termed C-P@ART and systematically characterized its physicochemical properties. Through *in vitro* and *in vivo* experiments, the antifibrotic actions of C-P@ART were validated, demonstrating its capacity to significantly ameliorate fibrosis by antagonizing TGF-β/SMAD and PI3K/Akt signaling pathways. Furthermore, this study demonstrated that C-P@ART inhibits the fibrotic process by suppressing the NF-κB signaling cascade, thereby modulating the aggregation of inflammatory cells. These findings provide new insights and a theoretical foundation for advancing antifibrotic therapies in ocular diseases.

## 2. Materials and methods

### 2.1. Chemicals and reagents

All reagents utilized are commercially available and verified by our suppliers. The ART solution was formulated by dissolving ART (Aladdin, #A107818-5g, Shanghai, China) in 5% sodium bicarbonate for *in vivo* subconjunctival injection at 30 mg/mL. In cellular experiments, ART (Solarbio, #88495-63-0, Beijing, China) was dissolved in DMSO at a concentration of 10 mM for preservation (–20 °C) and subsequently diluted in culture medium for fibroblast myofibroblast differentiation (FMD) experiments and cell death assessment. The injectable hydrogel was composed of natural type I collagen (Dubuwuqi Biomedical Technology Co., Ltd., Jiangsu, China) and four-armed polyethylene glycol *N*-hydroxysuccinimide (4-armed PEG-NHS, Xiamen Sinopeg Biotech Co., Ltd., Xiamen, China) as the carrier.

### 2.2. Chemical reagents preparation of C-P@ART

Natural type I collagen was dissolved in a 0.25 mol/L acetic acid solution and stirred overnight at room temperature to guarantee complete dissolution (Liang et al. 2026). The collagen solution was subsequently neutralized with sodium hydroxide to achieve a pH of 7–8, followed by vortexing and brief centrifugation. A 4 mg/mL collagen solution was formulated by diluting with phosphate-buffered saline (PBS) buffer to a fixed volume. This solution was stored at 4 °C and utilized as reaction solution A. Simultaneously, a 24 mg/mL solution of 4-armed PEG-NHS solution was prepared by mixing 4-armed PEG-NHS with ART solutions formulated in 5% sodium bicarbonate. The mixture was preserved as reaction solution B. The hydrogel was synthesized by mixing solutions A and B at a 3:1 ratio, yielding final concentrations of 3 mg/mL for natural type I collagen and 6 mg/mL for 4-armed PEG-NHS. After vortex mixing, brief centrifugation, and elimination of air bubbles, the hydrogel material was incubated at 37 °C for 15 min to facilitate gelation. Furthermore, to enable effective injection of hydrogel material into the subconjunctival tissue of animals, Reagents A and B were mixed and placed in a low-temperature environment of 4 °C to prolong the gelation time. Subsequently, a 1 mL syringe was utilized to effectively inject C-P@ART into the subconjunctival tissue around the eye.

### 2.3. Fourier-transform infrared (FT-IR)

The FT-IR spectrum curve of C-P@ART was obtained using the Bruker Tensor 27 spectrometer (Bruker, Germany) through the direct transmittance method based on the KBr pellet technique.

### 2.4. Drug release assay *in* *vitro*

To evaluate the release kinetics of the payload, 300 μL of C-P@ART hydrogel was immersed in 3 mL of PBS at 37 °C. At predetermined time intervals over a 24-h period, the supernatant was collected and replaced with an equal volume of fresh PBS to maintain sink conditions. The absorbance of each sample was measured at 221.5 nm using a UV-Vis spectrophotometer (UV-1900, Shimadzu, Japan). The ART concentration was subsequently determined according to a standard calibration curve, and ploted the cumulative release profile.

### 2.5. Rheological properties

The rheological properties of C-P@ART were examined using an MCR-92 rheometer (Anton Paar, Austria). Alternating strain variations (high-to-low strain scans: 1% → 400% → 1%) were applied at a fixed frequency (1 Hz) to examine the self-healing behavior of the C-P@ART, while the storage modulus (G′*G*′) and loss modulus (G″*G*″) were monitored. Strain sweep measurements were performed at a constant frequency of 1 Hz, with sequential strain changes from 1% to 400%, to evaluate shear-thinning behavior.

### 2.6. Scanning electron microscope (SEM)

The injectable hydrogel was synthesized by mixing collagen with an ART solution containing PEG-NHS, as described above. The microstructure of C-P@ART composite hydrogel after freeze-drying was observed utilizing SEM (TESCAN, Czech Republic).

### 2.7. Endotoxin-testing

Bacterial endotoxin levels in the hydrogel components were assessed using a Gel-Clot Tachypleus Amebocyte Lysate (TAL) assay (sensitivity *λ* = 0.5 EU/mL) (Zhanjiang Amoebocyte Lysate Plant, China). A total of 100 μL of each sample was mixed with an equal volume of TAL reagent in pyrogen-free tubes and incubated at 37 ± 1 °C for 60 ± 2 min. During incubation, tubes remained undisturbed to prevent mechanical disruption of the gel. Results were determined by gently inverting each tube 180 °. A positive result required the formation of a firm and intact gel. Endotoxin-free water and standard endotoxin solutions served as controls to validate the assay.

### 2.8. Primary human orbital fibroblast cells (OFs) culture

Samples were collected from the discarded periorbital connective tissue of patients undergoing strabismus correction surgery with muscle weakening procedures. The clinical data associated with all the sample sets have been completely de-identified, making it impossible to identify the patients' identities. This study was approved by the Clinical Research Ethics Committee of the Second Xiangya Hospital of Central South University (NO. LYEC2025-0212). The validity period of the ethically approved experiment is from 9 August 2025 to 19 August 2026. It is determined that the risk is no greater than the minimum risk, and thus meets the conditions for exemption from informed consent. All procedures followed the ethical standards established by the Ethics Committee and adhered to the principles of the Declaration of Helsinki (1975, revised in 2000).

The excised surgical specimens were immediately immersed in 4.5 g/L glucose-modified Dulbecco's modified Eagle medium (DMEM, Gibco, #C11965500BT, Waltham, MA, USA) supplemented with 20% fetal bovine serum (FBS, Bovogen, #SFBS, Australia), 1% penicillin‒streptomycin (Gibco, #15140-122, Waltham, MA, USA), and 1% L-glutamine (Gibco, #25030-081, Waltham, MA, USA). The specimens were preserved in a cell culture incubator at 37 °C with 5% CO_2_. The culture medium was changed every 3–5 days. OFs were passaged utilizing 0.25% trypsin-EDTA with phenol red (Gibco, #25200072, Waltham, MA, USA) upon achieving 80%–90%. After passaging, the concentration of fetal bovine serum in the medium was diminished from 20% to 10%. OFs from passages 3–6 (P3–P6) were utilized for subsequent experiments. Before use, fibroblasts were detected using immunofluorescence staining for vimentin.

### 2.9. Flow cytometry

OFs were seeded in 6-well plates at a density of 70–80% and cultured overnight prior to the indicated treatments. For drug-containing extract preparation, C-P@ART was thoroughly mixed and evenly spread on the base of a 6-well plate. After gelation, a 10-fold volume of complete medium was introduced and allowed to incubate for 24 h. The medium was subsequently collected as the drug-containing extract solution. After 24 h of treatment with drug-containing extracts, the cells were digested using 0.25% trypsin-EDTA for collection, followed by centrifugation at 1000 rpm for 5 min. The cells were subsequently washed twice with PBS, with each wash followed by centrifugation at 450 × g for 10 min. OFs were stained with propidium iodide (PI, 10 μM) to evaluate cell death. OFs were stained using a FITC-Annexin V/PI apoptosis detection kit (Multi-sciences, #AP100-100 kit, Hangzhou, China) to assess apoptosis. Stained cells were analyzed using flow cytometry (Cytek Bioenvironment, Northern Lights TM, USA). All experiments were performed in triplicate.

### 2.10. Cell viability assay

Primary cultured OFs were seeded into 96-well plates at 5 × 10^3^ cells per well. Before subsequent assays, the cells were incubated in 10-fold diluted extracts for 24 h. To assess cell viability, OFs were cultured in 96-well plates and exposed to varying concentrations of ART for 24 h, followed by detection using the Cell Counting Kit-8 (CCK-8) (Dojindo Laboratories, #CK04, Japan). The experiments were conducted at least five times. The half-maximal inhibitory concentration (IC_50_) was calculated utilizing GraphPad Prism software (GraphPad Software, San Diego, CA, USA).

### 2.11. Quantification of chemical release kinetics from hydrogels *in vivo*

Biotin-labeled ART was dissolved and mixed with 4-armed PEG-NHS and type I collagen to produce C-P@ART-biotin. Biotin was obtained from Xi'an Ruixi Biological Technology Co., Ltd. For immunofluorescence analysis, avidin (1:500, D-AKE21000, Biogradetech, CA, USA) was employed to conjugate with the biotin-labeled ART, followed by incubation with Alexa Fluor 488-labeled secondary reagents (Invitrogen, USA) for visualization. Cell nuclei were counterstained with DAPI. New Zealand rabbits were subsequently divided into two groups (*n* = 3–5 per group). One group received a subconjunctival injection of 100 μL ART-biotin solution, while the other group received a subconjunctival injection of C-P@ART-biotin. Injections were administered in one eye only, and the rabbits were euthanized on days 1, 3, and 7 after injection. Fascia tissue was harvested and fixed in paraffin. Hematoxylin and eosin (H/E) staining and immunofluorescence staining were performed to observe the residual distribution of ART in local subconjunctival tissues. Fluorescence intensity was assessed to quantify ART concentration in the fascia tissue, thereby reflecting the drug release profile *in vivo*.

### 2.12. Western blotting

After the specified treatment, cells were washed with PBS and lysed on ice utilizing radioimmunoprecipitation assay (RIPA) buffer. Protein concentrations were quantified utilizing a bicinchoninic acid (BCA) protein assay kit (Thermo, #23225, Massachusetts, USA) according to the manufacturer's instructions. Equal amounts of protein (50 μg) and 5 μL protein molecular weight markers (Thermo, #26616, Waltham, MA, USA) were separated using sodium dodecyl sulfate-polyacrylamide gel electrophoresis and transferred to polyvinylidene fluoride (PVDF) membranes (Pall Corporation, #BSP0161, New York, USA). The membranes were subsequently blocked with 5% bovine serum albumin (BSA) in PBS containing 0.1% Tween-20 and incubated with primary antibodies at 4°C overnight. After incubation, the PVDF membranes were washed thrice for 15 min each with PBS containing 0.1% Tween-20, followed by incubation with corresponding secondary antibodies at room temperature for 1 h. The membranes were subsequently washed thrice for 10 min each with PBS containing 0.1% Tween-20. The primary antibodies utilized in this study included *α*-SMA (Boster, #BM0002, Wuhan, China), SMAD2/3 (#5678), *p*-SMAD2/3 (#8828), Akt (#9272), *p*-Akt (#4056), NF-κB (#8242), *p*-NF-κB (#3033), procured from Cell Signaling Technology (Danvers, MA, USA), GAPDH (Abcam, #ab181602, Cambridge, UK), and *β*-Tubulin (Sino Biological, #100109-MM05T, Beijing, China). Protein bands were visualized using enhanced chemiluminescence (ECL) (Bio-Rad, ECL kit, California, USA). The relative band intensity was quantified using ImageJ software based on the digital peak area intensity to determine protein expression levels.

### 2.13. Immunofluorescence

Cells were seeded in 12-well plates and cultured for 24 h. Following incubation, cells were fixed with 4% paraformaldehyde for 15 min and permeabilized with 0.1% Triton X-100 in PBS for 10 min. After three washes with PBS, samples were blocked with 5% BSA in PBS for 30 min. Primary antibody incubation was performed using anti-*α*-SMA antibody (diluted in 5% BSA/PBS; anti-GFP 1:200, Boster, #BM0002, Wuhan, China) with 100 μl per coverslip overnight at 4 °C. Subsequently, cells were washed three times with PBS (10 min per wash) and blocked again with 5% BSA/PBS for 30 min. A Cy2-green conjugated goat anti-rabbit secondary antibody, coupled with AlexaFluor®555 fluorescent dye (Invitrogen, USA; anti-GFP 1:500), was applied and incubated for 1 h at room temperature. After six 5-min washes with PBS, the cells were counterstained with DAPI, and mounting medium was applied to glass slides. The fluorescence images were captured from eight fields of view of each cell coverslip, using a fluorescence microscope (Zeiss, Axio Imager M2, Germany). Quantification mean of the fluorescence intensity was performed for relevant analyzes.

### 2.14. Animals and groups of experiments

All animal experiments were approved by the Institutional Animal Care and Use Committee (IACUC) of the Second Xiangya Hospital, Central South University, China (NO. 20230512), and conducted in compliance with national and institutional guidelines provided by the American Veterinary Medical Association for the care and use of laboratory animals. The validity period of the ethically approved experiment was from 1 June 2023 to 1 June 2026. The animal license numbers was SYXK2022-0012.

To mitigate confounding hormonal fluctuations associated with the female estrous cycle, this study exclusively employed male New Zealand rabbits. This selection yielded a more homogeneous physiological cohort, thereby enhancing the precision and reproducibility of the experimental outcomes. Male New Zealand rabbits aged 3–5 months, weighing 2–2.5 kg, were included in this study. The rabbits were procured from the Laboratory Animal Center of the Second Xiangya Hospital, Central South University (Changsha, China), and allowed to acclimatize for 3–5 days before the experiments. New Zealand rabbits were anesthetized through intramuscular injection of 0.1 mL/kg thiazide hydrochloride (Dunhua Shengda Animal Drug Co., Ltd., Jilin, China) and 0.1 mL/kg Zoletil® (Virbac, Carros, France) at a 1:1 ratio. Topical ophthalmic anesthesia was attained utilizing 0.5% proparacaine hydrochloride eye drops (ALCAINE, Geneva, Switzerland). The depth of anesthesia was determined by observing the disappearance of corneal reflex and the absence of response when the rabbit's toes were pinched with forceps. During the experiment, the heart rate and breathing of the rabbits were continuously monitored to remain within the normal range, ensuring that the animals were in a suitable state of anesthesia without pain sensation throughout the entire surgical procedure. After the experiment was completed, the rabbits were euthanized in strict accordance with the guidelines for the humane killing of laboratory animals (GB/T39760-2021) by intravenous injection of excessive pentobarbital sodium (90 mg/kg). The breathing and heartbeat were repeatedly observed to have completely stopped, with dilated pupils and loss of corneal reflex.

The rabbits were randomly divided into three groups with 3–6 animals in each group: (1) the untreated group, which did not undergo surgery; (2) the negative control group, which underwent surgery and subconjunctival injection of 0.1 mL of 0.9% saline solution immediately after surgery; (3) the C-P@ART group, which received a subconjunctival injection of 0.1 mL of C-P@ART material containing 30 mg/mL ART immediately after surgery. All surgeries were performed on one eye only.

### 2.15. GFS model

A ‘7-shaped’ incision was made in the periorbital fascia at the supertemporal region of the rabbit eye. Subsequent to the incision, a conjunctival flap was created 5 mm above the corneal limbus, resulting in the limbus-based conjunctival flap. A rectangular scleral flap was carefully dissected and separated within the central region of the conjunctival flap area to ensure uniform thickness. The anterior chamber was accessed at the corneoscleral junction, and a peripheral iridectomy was performed. After establishing the outflow channel, the exposed fascial tissue was sutured; however, the scleral and conjunctival flaps were intentionally left unsutured to reduce excessive scarring.

### 2.16. Histological examination

The surrounding scleral tissue was excised from the vesicle and fixed with 4% PFA for at least 48 h. The sample was subsequently dehydrated and embedded in paraffin. Additionally, 4 µm thickness of continuous sections was performed for H/E, Masson, or IHC staining. The primary antibody utilized for IHC in this study was *α*-SMA (Boster, #BM0002, Wuhan, China).

### 2.17. Statistical analysis

GraphPad Prism software (Version 8, GraphPad, San Diego) was utilized for statistical analysis. Data are presented as mean ± standard deviation. All experiments were performed independently with at least three samples. Statistical significance was assessed using unpaired two-tailed *t*-tests for comparison of two groups. A one-way analysis of variance was used to compare multiple mean values. A *p* < 0.05 was considered statistically significant.

## 3. Results

### 3.1. Hydrogel preparation, characterization, and release kinetics of hydrogels *in vitro and in vivo*

As illustrated in Figure 1A, natural type I collagen, 4-armed PEG-NHS, and ART were mixed at pH 7–8 and incubated at 37 °C, resulting in a condensation reaction to form C-P@ART. Type I collagen comprises two α1 chains and one α2 chain, each containing approximately 1050 amino acid residues (Senadheera et al. 2020). As a monofunctional PEG derivative, 4-armed PEG-NHS possesses an active ester (NHS) group that reacts with the primary amine groups of collagen molecules, forming stable amide bonds at pH conditions of 7–8.5. PEG-activated esters are water-soluble and commonly used for modifying amino peptides, proteins, and various small molecules (Fernandes-Cunha et al. 2020; Say et al. 2024). The carboxyl group on the ART molecule binds to the active ester (NHS) group of the 4-armed PEG-NHS, forming a new stable ester bond that effectively encapsulates ART within the network structure formed by collagen and 4-armed PEG-NHS, leading to the formation of C-P@ART.

**Figure 1.**
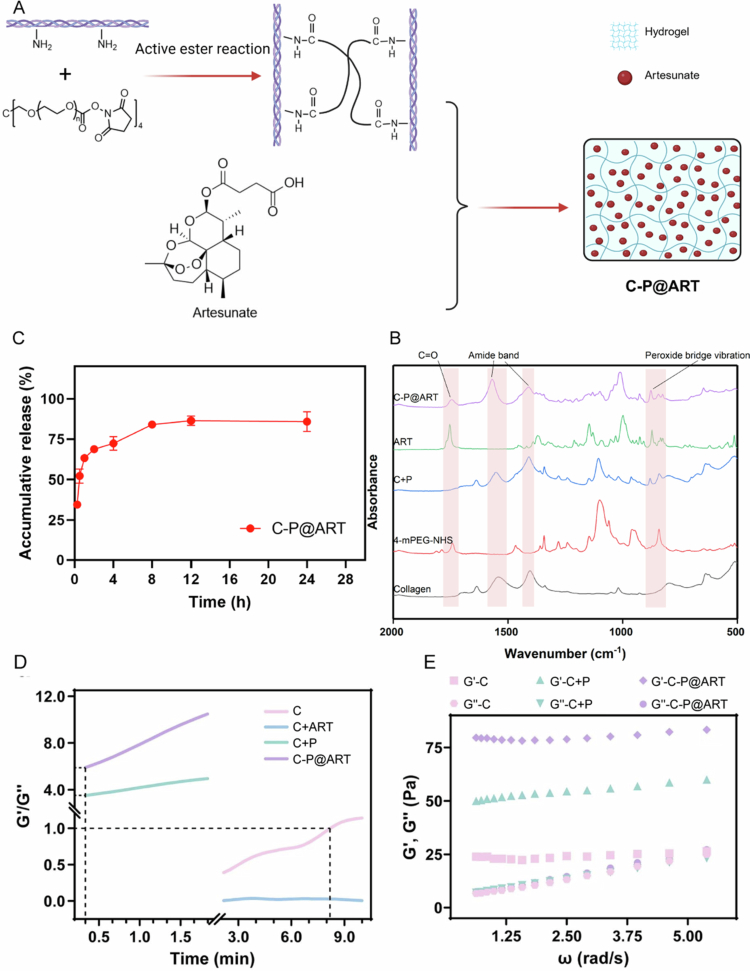
Preparation of C-P@ART sustained release hydrogel. (A) Schematic diagram for design and preparation of C-P@ART as an injectable hydrogel cross-linked by 4-armed PEG-NHS and collagen for localized sustained release of artesunate in postoperative fibrosis prevention after GFS. (B) cumulative percentage release graphs of ART from C-P@ART in vitro. (C) FT-IR spectrum curves of Collagen, 4-armed PEG-NHS, C + P, ART, and C-P@ART. (D) The storage (G′) and loss modulus (G″) of Collagen, C + ART, C + P, and C-P@ART formulation in the time-sweep test. (E) G′ and G″ of Collagen, C + P, and C-P@ART in the frequency-sweep experiment.

The successful preparation of the hydrogel and subsequent loading of ART were confirmed via FT-IR spectroscopy. In the C + P spectrum, the characteristic NHS ester absorption at 1740 cm^-^¹ almost disappeared, accompanied by the emergence of well-defined Amide I (~1650 cm^-^¹) and Amide II (~1550 cm^-^¹) bands, pointing to the creation of a stable cross-linked network between collagen and 4-armed PEG-NHS. For the C-P@ART formulation, the presence of ART-specific features, such as the C = O stretching at ~1750 cm^-^¹ and the distinctive fingerprint region between 800 and 1000 cm^-^¹, provides clear evidence of successful drug encapsulation. Most notably, the signature peak of the endoperoxide bridge at ~880 cm^-^¹, which represents the essential pharmacophore of ART, remained fully intact within the C-P@ART hydrogel. These findings collectively demonstrate that the structural integrity and pharmacological potency of ART were preserved throughout the encapsulation process.

As shown in Figure 1C, C-P@ART hydrogels exhibit specialized release kinetics characterized by a rapid initial phase followed by a high-level steady state. Approximately 70% of the ART is released within the first two hours, ensuring a robust therapeutic concentration that addresses the acute inflammatory response immediately following GFS. Crucially, the release profile transitions into a stable plateau phase from 8 h onward, maintaining consistent drug exposure throughout the 24-h observation period. Compared to the ultra-short biological half-life of native ART, this 24-h sustained presence significantly extends the effective window for antifibrotic intervention, ensuring a continuous inhibitory effect during the critical early stages of tissue healing.

Rheological analysis further confirmed the effective synthesis of C-P@ART. As illustrated in Figure 1D–E, the storage modulus (G′) of C + ART group was lower than the loss modulus (G″). However, the enhanced hydrogel sustained-release material (C-P@ART) demonstrated G′ and G″ values of approximately 80 and 25 Pa (G′ > G″), respectively, indicating the formation of a stable hydrogel. These results demonstrate the successful synthesis of the material. SEM was utilized to visualize the structure of the lyophilized C-P@ART in Figure S1. While the SEM images exhibit enlarged pores due to the expansion of ice crystals during freeze-drying, the hydrated network in physiological conditions provides a much denser structure.

The bacterial endotoxin test was performed to evaluate the biological safety of the C-P@ART hydrogel. As summarized in Table 1, the endotoxin levels of the Collagen, 4-armed PEG-NHS, and C + P groups were all below the detection limit of 0.5 EU/mL, consistently matching the negative control. These results confirm the non-pyrogenic nature and favorable biocompatibility of the synthesized materials, ensuring their suitability for intraocular implantation.

**Table 1.** Results of the semi-quantitative gel-clot endotoxin assay (*λ* = 0.5 EU/mL).

Group	Sample description	Replicate 1	Replicate 2	Replicate 3
NC	Endotoxin-free Water	−	−	−
PC	Standard Endotoxin (1.0 EU/mL)	+	+	+
Test 1	Collagen	−	−	−
Test 2	4-armed PEG-NHS	−	−	−
Test 3	C + P	−	−	−

*Table 1. TAL reagent sensitivity (λ) = 0.5 EU/mL.* ‘***+*** *’ indicates the formation of a firm gel that remains intact upon 180° inversion, representing an endotoxin concentration ≥0.5 EU/mL; ‘* ***−*** *’ indicates no gel formation or a fragile gel that breaks upon inversion, representing an endotoxin concentration <0.5 EU/mL. NC: Negative Control (Endotoxin-free water); PC: Positive Contro* *(* *Standard Endotoxin)*.

The cytocompatibility of C-P@ART was systematically assessed using CCK-8 assay and Calcein AM/PI dual-staining flow cytometry analysis in primary OFs. The dose–response analysis revealed an ART IC50 value of 410.3 μM (Figure 2C–D). The percentage of PI-positive cells demonstrated a concentration-dependent increase corresponding to ART dosage (Figure 2A–B). The blank hydrogel matrices exhibited favorable cytocompatibility, as evidenced by sustained cell viability above 90% across all experimental conditions. These findings indicate that the observed cytotoxic effects are specifically mediated by the pharmacological activity of ART rather than by inherent material toxicity from either the natural type I collagen or 4-armed PEG-NHS components.

**Figure 2.**
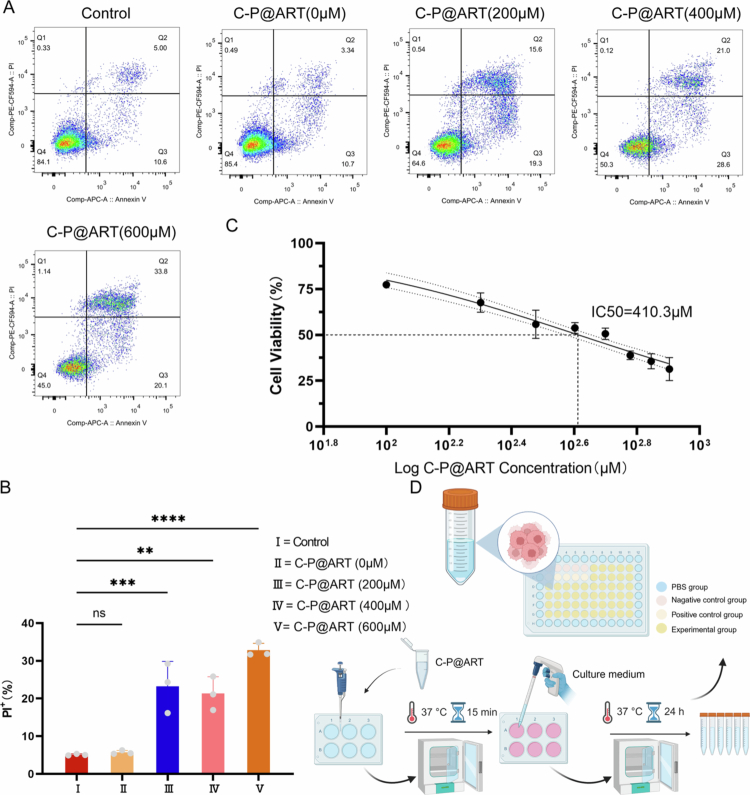
Characterization of C-P@ART sustained release hydrogel. (A-B) Cell death was measured using PI staining followed by flow cytometric analysis. (C) Cell viability was detected using the CCK-8 method. The semi-maximum inhibitory concentration IC_50_ (semi-inhibitory concentration) was measured using GraphPad Prism as 410.3 µM. Data are presented as mean ± standard deviation (SD), and one-way analysis of variance (ANOVA) was used for statistical analysis. *p < 0.05, **p < 0.01, ***p < 0.001, ****p < 0.0001. (D) Schematic diagram for detection of cell viability using the CCK-8 method.

Given the favorable biocompatibility of C-P@ART and the limitations of *in vitro* models in fully replicating the complex postoperative microenvironment of GFS, biotin labeling was employed to further investigate the *in vivo* drug release kinetics. We labeled ART with biotin (Figure 3A) to evaluate the *in vivo* release profile of C-P@ART. As illustrated in Figure 3B, on day 1 after subconjunctival injection, ART-biotin was uniformly distributed across the sclera and subscleral region, with no significant difference in mean fluorescence intensity observed between ART-biotin and C-P@ART-biotin groups (16,651,415 ± 3,639,191 versus 13,384,695 ± 4,724,495, *p* = 0.3965). On days 3 and 7 after injection (Figure 3C–D), ART-biotin was predominantly localized in the subscleral region, with the average fluorescence intensity of the C-P@ART-biotin group significantly higher than that of the ART-biotin group (38,176,688 ± 4,855,628 versus 16,149,405 ± 2,932,384, *p = 0.0482 for day 3; 18,305,005 ± 4,184,940 versus 11,706,731 ± 1,806,896, *p = 0.0344 for day 7). The differences in fluorescence intensity gradually decreased over time. These results revealed that C-P@ART prolongs the action time of ART through sustained release.

**Figure 3.**
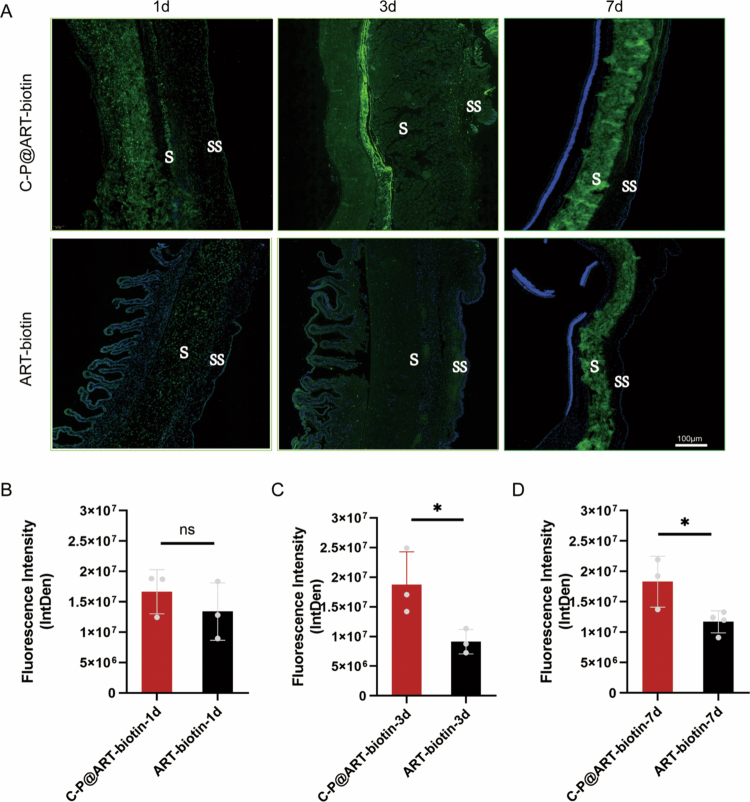
Quantification of release kinetics from hydrogels *in vivo*. (A) ART was combined with biotin and injected into subconjunctiva alone or wrapped in a slow-release material. (B-D)The fluorescence intensity of ART-biotin in conjunctiva was analyzed after days 1, 3, and 7 after surgery to reflect the release and degradation of the drug *in vivo*. C, conjunctiva; SS, subconjunctival space; S, sclera. Scale bars = 100 µm. Data are presented as mean ± SD, and Student's t-test was used for statistical analysis. *p < 0.05, **p < 0.01, ***p < 0.001, ****p < 0.0001.

### 3.2. C-P@ART inhibits TGF-β1-induced fibroblast-myofibroblast differentiation by blocking SMAD and non-SMAD pathways in OFs

TGF-β1-induced OFs *in vitro* fibrotic model was utilized to investigate the anti-fibrosis efficacy *in vitro*. The drug-containing extracts were administered in OFs as follows: the untreated control group, the TGF-β1-induced group, TGF-β1-induced groups treatedwith 0, 200, 400, or 600 μM C-P@ART, respectively. The expression levels of *α*-SMA protein in each group were analyzed using Western blotting and Immunofluorescence. The results are illustrated in Figure 4C-D that compared to the untreated control group, TGF-β1 induction significantly increased *α*-SMA expression by 39% (1.390 ± 0.14 versus 1.000 ± 0.00, ****p = 0.0004). However, treatment with a C-P@ART extraction solution resulted in a concentration-dependent inhibition of *α*-SMA expression. Compared to the TGF-β1-induced group, *α*-SMA levels were reduced to 0.844 ± 0.15 (200 μM, ****p < 0.0001), 0.737 ± 0.06 (400 μM, ****p < 0.0001), and 0.586 ± 0.10 (600  μM, ****p < 0.0001), respectively. The blank hydrogel extract did not exhibit significant therapeutic effects (TGF-β1 + C-P@ART (0 μM) group 1.383 ± 0.22 versus TGF-β1 group 1.390 ± 0.14, *p* = 0.9999), further confirming the specific bioactivity of ART in C-P@ART. Likewise, immunofluorescence analysis showed that TGF-β1-induced *α*-SMA expression was reduced by C-P@ART (Figure 4H-I).

**Figure 4.**
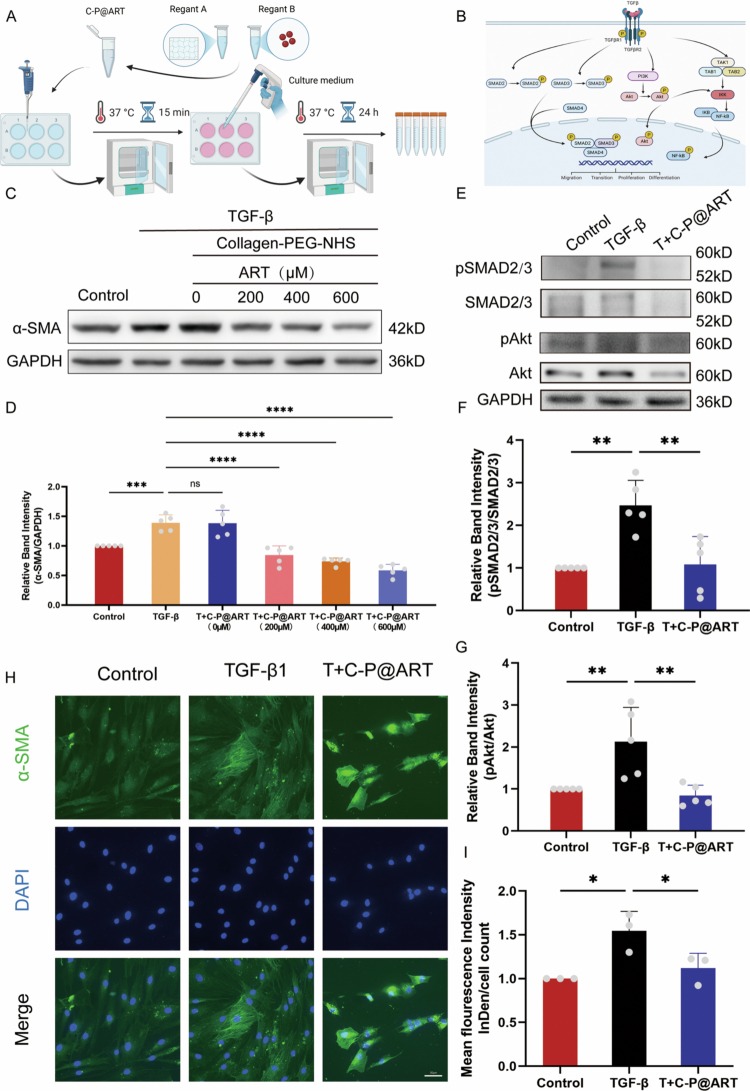
*The release of ART from C-P@ART sustained-release material down-inhibits α-SMA expression and phosphorylation of SMAD2/3 and Akt in TGF-* *β* *1-induced OFs. (* *A* *)* *Illustration of the extraction process of the drug-containing extract*. *(* *B* *)* *The schematic diagram of TGF-β1 activating the classical SMAD2/3–SMAD4 signaling pathway and the non-classical signaling pathways.* *The expressions of (* *C* *-* *D* *)* *α* *-SMA, (* *E* *-* *G* *) p-SMAD/SMAD*, *and p-Akt/Akt in primary OFs treated with 10ng/L TGF-β1 alone or co-treated with C-P@ART slow-release material extracts containing different concentrations of ART were analyzed* *using* *western blotting.* *GAPDH served as the loading control.* *The expressions of (* *H* *-* *I* *)* *α* *-SMA in primary OFs treated with 10ng/L TGF-β1 alone or co-treated with C-P@ART were analyzed* *using* *immunofluorescence.* *Scale b* *ar* *=* *50* *μm* *.* *Data are represented as mean* *±* SD of 3–5 independent experiments. One-way ANOVA was used for statistical analysis. *p < *0* .05, **p < *0* .01, ***p < *0* *.001* *,* ****p < *0*.*0* *001*.

Furthermore, TGF-β1 functions as a master regulator through activation of both canonical SMAD2/3–SMAD4 signaling and noncanonical pathways (Figure 4B). The expression levels of *p*-SMAD/SMAD and *p*-Akt/Akt in OFs were analyzed after 24 h of extract treatment in TGF-*β*-induced OFs to evaluate the effect of C-P@ART on the TGF-*β* pathway. The results revealed that, compared to the untreated control group, TGF-β1 stimulation significantly activated the SMAD2/3 phosphorylation (2.472 ± 0.58 versus 1.000 ± 0.00, **p = 0.0012) and Akt (2.125 ± 0.82 versus 1.000 ± 0.00, **p = 0.0031). However, C-P@ART treatment largely reversed these effects, reducing SMAD2/3 phosphorylation to 1.081 ± 0.66 (**p = 0.0019 versus TGF-β1 group) and Akt phosphorylation to 0.842 ± 0.25 (**p = 0.0041 versus TGF-β1 group) (Figure 4E-D). These results revealed that C-P@ART can inhibit fibrosis in OFs through the classical SMAD signaling pathway and the PI3K/Akt signaling axis.

### 3.3. C-P@ART inhibits fibroblast-myofibroblast differentiation and fibrosis *in vivo*

We initially assessed local and systemic reactions in rabbit models on days 1 and 28 postoperative intervals. Experimental (surgery + C-P@ART) and control (surgery + Collagen-PEG-NHS) groups exhibited similar physiological responses. As illustrated in Figure 5A, transient ocular manifestations, including mild conjunctival hyperemia, minor subconjunctival hemorrhage, and temporary corneal edema, were observed. H/E staining illustrated in Figure 5B–E revealed that C-P@ART exhibited no significant toxicity compared with the collagen-PEG-NHS group in the cornea, subconjunctival, ciliary body, and retina. Furthermore, all animals exhibited standard behavioral patterns (alertness, mobility), circadian rhythms, feeding habits, and survival rates (100%). Subconjunctival administration of C-P@ART demonstrated excellent biosafety profiles, with no evidence of acute/chronic local or systemic toxicity, thereby confirming its biocompatibility for ophthalmic applications.

**Figure 5.**
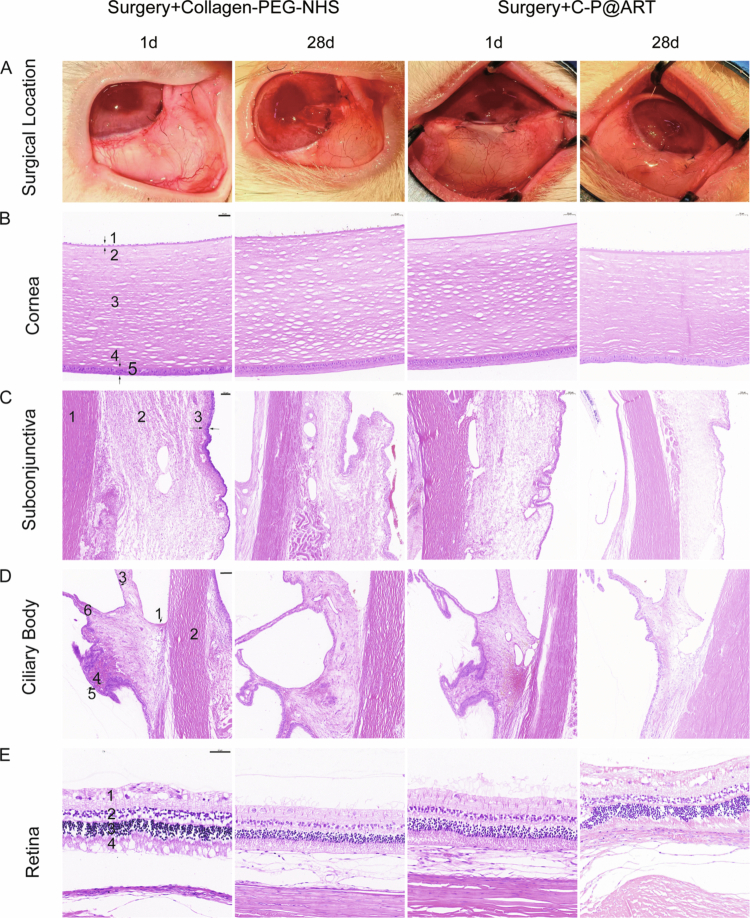
C-P@ART demonstrated excellent biosafety profiles and biocompatibility in rabbit glaucoma filtering surgery models. Rabbits are randomly assigned into surgery + Collagen-PEG-NHS and surgery + C-P@ART groups. Rabbits were euthanized on day 1 or 28 after surgery. (A) The external appearance of transient ocular manifestations of rabbits in two groups. (B) H/E staining at the cornea of surgical sites of rabbits in two groups. 1, corneal endothelium; 2, posterior boundary layer; 3, corneal stroma; 4, anterior boundary layer; 5, corneal epithelium. Scale bars = 50 µm. (C) H/E staining at the subconjunctival of surgical sites of rabbits in two groups. 1, sclera; 2, subconjunctival space; 3, conjunctiva. Scale bars = 100 µm. (D) H/E staining at the ciliary body of surgical sites of rabbits in two groups. 1, anterior chamber angle formed by iris and trabecular mesh; 2, sclera; 3, pigment epithelium of the iris; 4, non-pigment cells; 5, pigment cells; 6, ciliary process. Scale bars = 100 µm. (E) H/E staining at the retina of surgical sites of rabbits in two groups. 1, ganglion cells; 2, bipolar cells; 3, optic cells; 4, pigment epithelial cells. Scale bars = 50 µm.

The *in vivo* anti-fibrotic efficacy of C-P@ART was assessed. We administered saline (100 μL) or C-P@ART material containing 30 mg/mL ART (100 μL) subconjunctivally at the surgical site immediately after surgery in a GFS model. A surgical group served as the control. Fascial tissues from the surgical site were collected for H/E staining, Masson's trichrome staining, and *α*-SMA IHC analysis on day 28 after surgery (Figure 6A–B).

**Figure 6.**
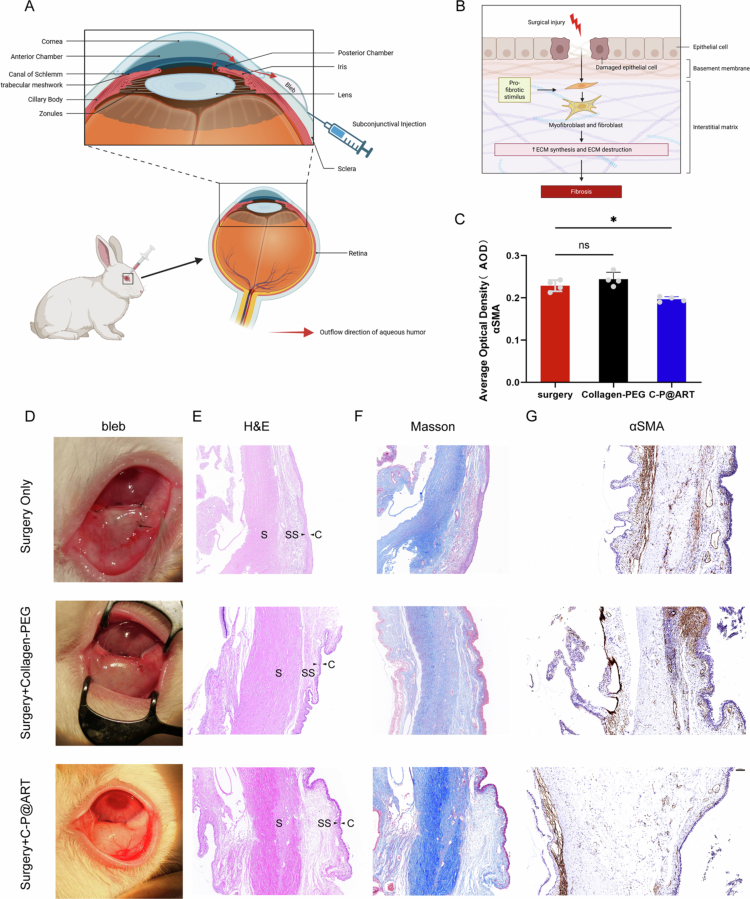
*C-P@ART* *inhibited* *subconjunctival fibrosis in rabbit glaucoma filtering surgery model. Rabbits* *were* *randomly assigned into three groups: surgery-only, surgery + Collagen-PEG-NHS, and surgery + C-P@ART groups* *;* *r* *abbits were euthanized on* *day 28 after surgery* *. (A)* *The* *s* *chematic* *diagram* *for ocular structure and outflow direction of aqueous humor after localized injection of C-P@ART after GFS. (B)* *The* *s* *chematic* *diagram* *for fibrosis process after surgical injury. (C)* *The quantitative analysis of* *α* *-SMA immunohistochemical staining at surgical sites of* *rabbit’s* *eyes in three groups. (D)* *The* *e* *xternal appearance of filtering blebs of rabbits in three groups. (* *E* *)* *H* */* *E staining at surgical sites of rabbits in three groups. C, conjunctiva; SS, subconjunctival space; S, sclera. (* *F* *)* *Masson staining of collagen organization and density at surgical sites of rabbits in three groups. (* *G* *)* *Immunohistochemical staining at surgical sites of rabbits in three groups. Scale bars* *= 200* *µ* *m. Data are* *presented* *as* *mean* *±* *SD* *.* *One-way ANOVA* was used for statistical analysis. *p *<* 0.05, **p *<* *0.01,* ***p < *0* *.001* *,* ****p < *0*.*0* *001*.

Figure 6D illustrates that compared to the surgical-only group, the C-P@ART-treated group displayed elevated and larger blebs with a diffuse distribution, signifying functional blebs and successful surgical outcomes. Figure 6E–F illustrates that H/E and Masson's staining of local surgical tissues revealed that subconjunctival fibrosis occurred normally in the hydrogel material group, with no significant inhibition or enhancement of fibrosis compared to the surgical-only group. However, subconjunctival tissues treated with C-P@ART exhibited greater tissue looseness and reduced postoperative collagen deposition, indicating decreased subconjunctival fibrosis.

Furthermore, *α*-SMA is the hallmark of activated fibroblasts transitioning into myofibroblasts, which are responsible for the excessive production of ECM that characterizes fibrosis. As illustrated in Figure 6C and G, the C-P@ART-treated group exhibited reduced *α*-SMA expression (0.2289 ± 0.01 versus 0.1967 ± 0.01, *p = 0.011) than the surgical-only group. No significant difference was observed between the hydrogel material group and the surgical-only group (0.2289 ± 0.01 versus 0.2440 ± 0.02, *p* = 0.21). Collectively, these results revealed that our C-P@ART can produce stable and long-lasting anti-fibrotic effects over an extended period with stable biocompatibility.

### 3.4. *In vitro* and *in vivo* effect of inflammatory suppression of C-P@ART

The fibrotic cascade commences with inflammatory activation, during which recruited immune cells secrete profibrotic mediators that stimulate fibroblast differentiation and perpetuate tissue injury. We systematically analyzed the activation of the NF-κB signaling pathway by monitoring p65 subunit phosphorylation to clarify the antifibrotic mechanism of C-P@ART through inflammatory modulation. Western blotting revealed that TGF-β1 stimulation significantly upregulated p65 phosphorylation levels compared to untreated controls (1.563 ± 0.26 versus 1.000 ± 0.00, **p = 0.0011) in OFs. Notably, intervention with C-P@ART extract substantially attenuated the p65 subunit phosphorylation-mediated inflammatory activation post-surgery (0.554 ± 0.20 versus TGF-β1 group 1.563 ± 0.26, ****p < 0.001) (Figure 7A–B).

**Figure 7.**
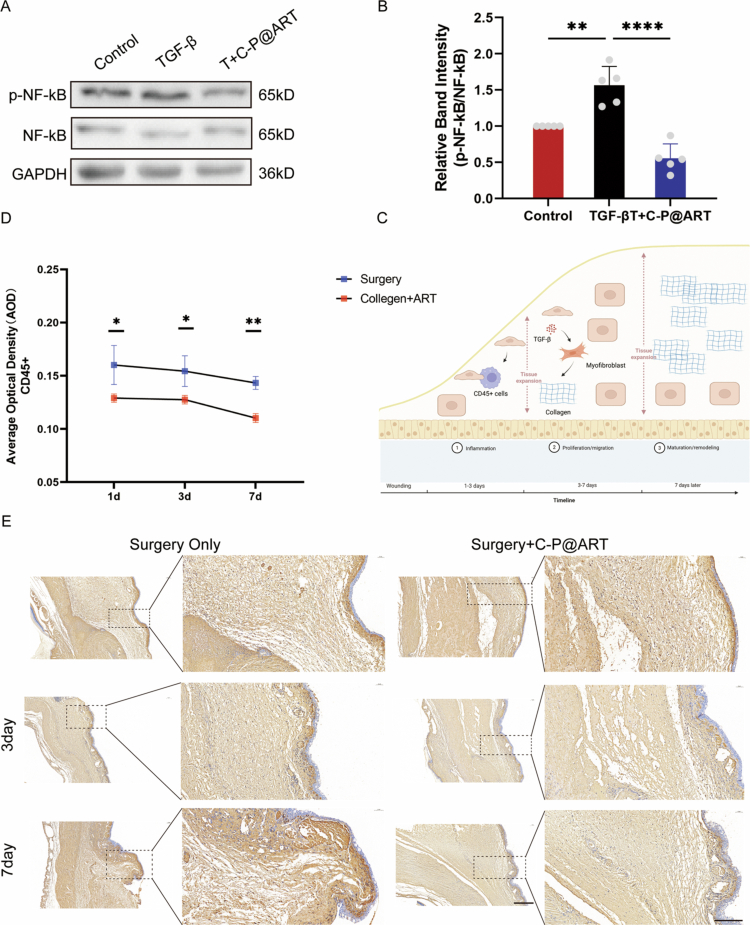
The C-P@ART released ART to inhibit GFS-induced inflammation. Western blot analysis (A) and statistical analysis (B) of the *p*-NF-kB/NF-kB expression in primary OFs control, primary OFs treated with 10 ng/L TGF-β1 alone, with 10 ng/L TGF-β1 + C-P@ART extract. GAPDH served as the loading control. (C) The schematic diagram for inflammation reaction in fibrosis process after surgical injury. (D, E) The quantitative analysis of CD45 + immunohistochemical staining at surgical sites of rabbit's eyes in each group. Rabbits were randomly divided into the surgery group and surgery + C-P@ART group and were euthanized on day 28 after surgery. The conjunctival tissue was taken from the surgical site for the immunohistochemical staining of CD45 and quantitative analysis in rabbits. Left panels: Scale bar = 100 μm. Right panels: Scale bar = 50 μm. The relative band intensity was quantified using ImageJ software. Data are presented as mean ± SD of three to five independent experiments. One-way ANOVA was used for statistical analysis. *p < 0.05, **p < 0.01, ***p < 0.001, ****p < 0.0001.

Consistent with *in vitro* findings, longitudinal tracking of leukocyte infiltration through CD45 immunomarker revealed temporal modulation of inflammation of C-P@ART treatment (Figure 7C). While no significant intergroup differences were observed at early time points (day 1: 0.1601 ± 0.02 versus 0.1290 ± 0.01, *p = 0.045; day 3: 0.1526 ± 0.01 versus 0.1223 ± 0.01, *p = 0.036), C-P@ART treatment exhibited progressive anti-inflammatory efficacy. A significant reduction in CD45 expression was achieved in the treatment group compared to the surgery-only controls (0.1432 ± 0.01 versus 0. 1103 ± 0.01, **p = 0.0015) on day 7 after surgery (Figure 7D–E). Collectively, these results revealed that C-P@ART could significantly inhibit the infiltration of inflammatory cells after surgery.

## 4. Discussion

Recently, various materials, including nanoparticles and gels, have been extensively utilized in the development of drug delivery systems for sustained release (Tao et al. 2022; Zhou et al. 2022). These materials facilitate the slow release of medications by physically adsorbing them onto the surfaces of polymer carriers or chemically encapsulating them within the porous structures of carrier materials through different covalent bonds. These sustained-release systems prolong the release time of drugs to enhance therapeutic efficacy and reduce drug toxicity and side effects through their biodegradable properties (Caja et al. 2018). Injectable hydrogels have demonstrated significant clinical potential in ocular disease treatment, especially those affecting the posterior segment of the eye (Ji et al. 2024; Liu et al. 2024b). Employing temperature-sensitive or pH-sensitive hydrogels to create carrier structures with appropriate pore sizes enables the encapsulation and *in situ* injection of medications into specific ocular tissues, including subconjunctival and subretinal regions, significantly improving local drug bioavailability.

Our previous studies have demonstrated that ART can effectively inhibit postoperative scar formation in glaucoma (Liu et al. 2023; 2024a). However, the application of ART is limited by its poor solubility, short half-life, and rapid metabolism. Hence, we aimed to develop a temperature-sensitive and pH-sensitive composite injectable hydrogel suitable for subconjunctival injection after GFS to overcome these limitations. This material was synthesized by conjugating 4-armed PEG-NHS with natural type I collagen, resulting in a network-like porous structure for encapsulating ART. We further systematically evaluated the characteristics of this injectable hydrogel, including surface morphology and rheological properties, and confirmed its injectability and gelation stability for subconjunctival administration. The results revealed that it completely meets the criteria for drug loading and release as a sustained-release delivery system. Furthermore, the examination of local drug retention in the conjunctiva validated the excellent sustained-release performance of this material. Cytocompatibility assays and animal toxicity studies confirmed that the hydrogel exhibited no significant toxic effects. All the above highlights its superior capability for ART delivery.

*In vitro* and *in vivo* findings confirmed the antifibrotic effects of the injectable hydrogel. The injectable hydrogel significantly inhibited the proliferation and differentiation of fibrosis-related cells on crucial fibrotic signaling pathways, including TGF-β1/SMAD and PI3K/Akt pathways. The injectable hydrogel exerts anti-inflammatory effects by regulating the NF-κB signaling pathway, significantly reducing inflammatory CD45 +  myeloid cell aggregation and thereby effectively mitigating the fibrotic process. Immune cell infiltration is essential for fibrosis. Within the first day postinjury, inflammatory cells rapidly accumulate at the injury site, including macrophages and neutrophils. On day 3, these cells further proliferate and differentiate, releasing several fibroblast-promoting factors and inflammatory cytokines while simultaneously activating various immune cells, thereby exacerbating tissue damage (Eming et al. 2014; Lombardi et al. 2019). These data enhance the potential application of our material as a multifunctional hydrogel in ocular medicine.

The innovation of this study lies in the design and development of an injectable hydrogel-based sustained-release medication delivery system for ART. The research on antifibrosis materials is primarily through physical action, including nanomaterials, which is limited to clinical transformation (Li et al. 2025). Our hydrogel system can be injected locally into the subconjunctival space, forming a gel *in situ,* facilitating the sustained release of ART and its continuous antifibrotic effects. Given that the scarring process in the glaucoma filtration pathway is a long-term pathological development, the sustained release of ART can effectively prolong its therapeutic duration and significantly improve treatment outcomes. Furthermore, the material exhibits good biocompatibility and will not induce toxicity and side effects on the local conjunctival tissue or the entire organism. Consequently, the injectable hydrogel system developed in this study addresses the pharmacokinetic limitations of ART and also provides essential clinical translational value for improving the success rate of GFS while ensuring safety.

This study has some limitations. First, regarding clinical translation, although our hydrogel carrier material exhibits good biocompatibility and safety, the 4-armed PEG-NHS utilized in the preparation process is a chemically synthesized product without sufficient clinical safety data. Consequently, subsequent studies should investigate alternative materials or preparation processes that meet clinical safety standards. Second, the specific mechanisms of ART in ocular antifibrosis require further comprehensive investigation. There is cross-regulation between NF-κB and TGF-β/Smad signaling pathways: TGF-β1 regulates the transcription of Smad7 through the direct binding of Smad3 and Smad4 to the Smad7 promoter (Liu et al. 2013), while Smad7 induces IκBα expression, an inhibitor of NF-κB, thereby inhibiting NF-κB-dependent inflammatory responses and modulating the inhibitory effects of TGF-β1 on IL-1β-induced inflammatory responses in macrophages (Hu et al. 2021). These mechanisms offer new research directions for investigating the antifibrotic effects of ART.

## 5. Conclusion

This study successfully developed a new sustained-release hydrogel material (C-P@ART) for stable loading and sustained ART release. *In vitro* and *in vivo* experiments revealed that C-P@ART reduced fibroblast activation by inhibiting TGF-β1/SMAD and PI3K/Akt pathways while exerting anti-inflammatory effects by suppressing the NF-κB pathway and inflammatory cell infiltration. Compared to existing antifibrotic therapies, the sustained-release characteristics of C-P@ART mitigate the limitations of ART's poor solubility and short half-life, targeting acute and chronic phases of subconjunctival fibrosis, thus providing a promising strategy to enhance GFS outcomes. This study offers new insights and theoretical foundations for antifibrotic therapy following glaucoma filtration surgery, which offers a new drug delivery system for clinical application. The findings are expected to promote additional investigation of injectable hydrogels in ocular disease treatment and offer new research directions for therapeutic strategies in glaucoma and other ocular diseases.

## Supplementary Material

Figure captions.docx Figure captions.docx

FIGURE S1.tif FIGURE S1.tif

## Data Availability

The original data presented in this study are available from the corresponding author on reasonable request.
