# Supplementary material for: An injectable hydrogel for localized sustained release of artesunate in postoperative fibrosis prevention after glaucoma filtration surgery
Source: Drug Deliv. 2026 Aug 3;33(1):2686511. doi: 10.1080/10717544.2026.2686511 (PMC13435280; doi:10.1080/10717544.2026.2686511)
Supplement: Supplementary file 1 [file IDRD_A_2686511_SM6225.docx]

Figure 1. *Preparation of C-P@ART sustained release hydrogel. (A)* *Schematic diagram for design and preparation of C-P@ART as an injectable hydrogel crosslinked by 4-armed PEG-NHS and collagen for localized sustained release of artesunate in postoperative fibrosis prevention after GFS. (B) cumulative percentage release graphs of ART from C-P@ART in vitro. (C) FT-IR spectrum curves of Collagen, 4-armed PEG-NHS, C+P, ART, and C-P@ART. (D) The storage (G’) and loss modulus (G”) of Collagen, C+ART, C+P, and C-P@ART formulation in the time-sweep test. (E) G’ and G” of Collagen, C+P, and C-P@ART in the frequency-sweep experiment.*

**Figure S1.** *Morphologies and size distribution of lyophilized C-P@ART captured by SEM. Left panel: Scale bar = 500 μm. Middle panel: Scale bar = 100 μm. Right panel: Scale bar = 10 μm.*

**Figure 2**. *Characterization of C-P@ART sustained release hydrogel. (A-B)* *Cell death was measured using PI staining followed by flow cytometric analysis. (C)* *Cell viability was detected using the CCK-8 method. The semi-maximum inhibitory concentration IC_50_ (semi-inhibitory concentration) was measured using GraphPad Prism as 410.3 µM. Data are presented as mean ± standard deviation (SD),* *and one-way analysis of variance (ANOVA)* *was used for statistical analysis. *p < 0.05, **p < 0.01, ***p < 0.001, ****p < 0.0001. (D) Schematic diagram for detection of cell viability using the CCK-8 method.*

**Figure 3.** *Quantification of release kinetics from hydrogels in vivo. (A-B)* *ART was combined with biotin and injected into subconjunctiva alone or wrapped in a slow-release material. The fluorescence intensity of ART-biotin in conjunctiva was analyzed after days 1, 3, and 7 after surgery to reflect the release and degradation of the drug in vivo. C, conjunctiva; SS, subconjunctival space; S, sclera. Scale bars = 100 µm. Data are presented as mean ± SD, and Student's t-test was used for statistical analysis. *p < 0.05, **p < 0.01, ***p < 0.001, ****p < 0.0001.*

**Figure 4**. *The release of ART from C-P@ART sustained-release material down-inhibits α-SMA expression and phosphorylation of SMAD2/3 and Akt in TGF-β1-induced OFs. The expressions of (A-B) α-SMA, (C-E) p-SMAD/SMAD, and p-Akt/Akt in primary OFs treated with 10ng/L TGF-β1 alone or co-treated with C-P@ART slow-release material extracts containing different concentrations of ART were analyzed using western blotting. GAPDH served as the loading control. The expressions of (F-G) α-SMA in primary OFs treated with 10ng/L TGF-β1 alone or co-treated with C-P@ART were analyzed using immunofluorescence. Scale bar = 50 μm. Data are represented as mean ± SD of 3–5 independent experiments. One-way ANOVA was used for statistical analysis. *p < 0.05, **p < 0.01, ***p < 0.001, ****p < 0.0001.*

**Figure 5.** *C-P@ART demonstrated excellent biosafety profiles and biocompatibility in rabbit glaucoma filtering surgery models. Rabbits are randomly assigned into surgery + Collagen-PEG-NHS and surgery + C-P@ART groups. Rabbits were euthanized on day 1 or 28 after surgery. (A)* *The external appearance of* *transient ocular manifestations of rabbits in two groups. (B)* *H/E staining at the cornea of surgical sites of rabbits in two groups. 1, corneal endothelium; 2, posterior boundary layer; 3, corneal stroma; 4, anterior boundary layer; 5, corneal epithelium. Scale bars = 50 µm. (C) H/E staining at the subconjunctival of surgical sites of rabbits in two groups. 1, sclera; 2, subconjunctival space; 3, conjunctiva. Scale bars = 100 µm. (D) H/E staining at the ciliary body of surgical sites of rabbits in two groups. 1, anterior chamber angle formed by iris and trabecular mesh; 2, sclera; 3, pigment epithelium of the iris; 4, non-pigment cells; 5, pigment cells; 6, ciliary process. Scale bars = 100 µm. (E) H/E staining at the retina of surgical sites of rabbits in two groups. 1, ganglion cells; 2, bipolar cells; 3, optic cells; 4, pigment epithelial cells. Scale bars = 50 µm.*

**Figure 6.** *C-P@ART inhibited subconjunctival fibrosis in rabbit glaucoma filtering surgery model. Rabbits were randomly assigned into three groups: surgery-only, surgery + Collagen-PEG-NHS, and surgery + C-P@ART groups; rabbits were euthanized on day 28 after surgery. (A)* *The schematic diagram for ocular structure and outflow direction of aqueous humor after localized injection of C-P@ART after GFS. (B)* *The schematic diagram for fibrosis process after surgical injury. (C)* *The quantitative analysis of α-SMA immunohistochemical staining at surgical sites of rabbit’s eyes in three groups. (D)* *The external appearance of filtering blebs of rabbits in three groups. (F)* *H/E staining at surgical sites of rabbits in three groups. C, conjunctiva; SS, subconjunctival space; S, sclera. (G)* *Masson staining of collagen organization and density at surgical sites of rabbits in three groups. (I)* *Immunohistochemical staining at surgical sites of rabbits in three groups. Scale bars = 200 µm. Data are presented as mean ± SD. One-way ANOVA was used for statistical analysis. *p < 0.05, **p < 0.01, ***p < 0.001, ****p < 0.0001.*

**Figure 7.** *The C-P@ART released ART to inhibit GFS-induced inflammation. Western blot analysis (A) and statistical analysis (B) of the p-NF-kB/ NF-kB expression in primary OFs control, primary OFs treated with 10 ng/L TGF-β1 alone, with 10 ng/L TGF-β1 + C-P@ART extract. GAPDH served as the loading control. (C)* *The schematic diagram for inflammation reaction in fibrosis process after surgical injury. (D, E) The quantitative analysis of CD45+ immunohistochemical staining at surgical sites of rabbit’s eyes in each group. Rabbits were randomly divided into the surgery group and surgery + C-P@ART group and were euthanized on day 28 after surgery. The conjunctival tissue was taken from the surgical site for the immunohistochemical staining of CD45 and quantitative analysis in rabbits. Left panels: Scale bar = 100 μm. Right panels: Scale bar = 50 μm. The relative band intensity was quantified using ImageJ software. Data are presented as mean ± SD of three to five independent experiments. One-way ANOVA was used for statistical analysis. *p < 0.05, **p < 0.01, ***p < 0.001, ****p < 0.0001.*
